# Peripheral pure ground-glass opacity: segmentectomy versus wedge resection

**DOI:** 10.1186/s13019-021-01610-y

**Published:** 2021-09-14

**Authors:** Taobo Luo, Qixun Chen, Jian Zeng, Lei Cai, Xiancong Huang

**Affiliations:** 1 Department of Thoracic Surgery, Cancer Hospital of The University of Chinese Academy of Sciences (Zhejiang Cancer Hospital), 1# East Banshan Road, Hangzhou, 310022 Zhejiang China; 2 Institute of Cancer and Basic Medicine (IBMC), Chinese Academy of Sciences, Hangzhou, Zhejiang China

**Keywords:** Peripheral ground-glass opacity, Segmentectomy, Wedge resection, Surface area

## Abstract

**Introduction:**

Sublobar resection has been widely accepted for treating pure ground-glass opacities (GGOs). As GGOs have good prognosis, preserving postoperative pulmonary function is the major concern in surgery. No studies have yet compared the success rates of pulmonary function reservation between segmentectomy and wedge resection.

**Method:**

The three-dimensional rebuild of computed tomography (CT) images was performed, the segmentectomy and wedge resection of the GGO in the target segment were simulated, and the area of cut surface was measured, which was important data for successful postoperative pulmonary recruitment maneuvers.

**Result:**

With equal volumes of tissue removed, segmentectomy and wedge resection showed similar surface area loss for RS4 and RS5, followed by LS7 + 8, LS6 and LS1 + 2 segments. Compared with other segments, wedge resection performed in RS10, LS3, LS10, RS9 and RS7 may lead to a loss of lot more surface area than segmentectomy.

**Conclusion:**

Wedge resection is suggested for segments RS4, RS5, LS1 + 2 and LS7 + 8, whereas segmentectomy is advised for segments RS1, LS4 + 5 and RS2. Meanwhile, deep wedge resection should be avoided for segments RS8, RS7, RS10, LS3, LS10. RS9 and LS9, in order to preserve a larger lung surface area.

## Introduction

With the increasing acceptance of routine computed tomography (CT) screenings, early-stage lung cancer detection is also becoming more frequent. Ground-glass opacities or GGOs are among lung cancers [1]. The persistent presence of a GGO nodule usually suggests the diagnosis of atypical adenomatous hyperplasia (AAH), adenocarcinoma in situ (AIS), or malignancies such as minimally invasive adenocarcinoma (MIA), or lepidic-predominant invasive adenocarcinomas (LPA) [2, 3]. Pathological diagnosis is highly correlated with imaging manifestations [4], and the management of GGO depends on the overall size and existence of a solid component [5].

Surgical excision is optional for pure GGOs up to 5 mm if they are increasing in size and for pure GGOs larger than 10 mm that remain stable but are persistent [6]. The surgical strategy for treatment, however, remains controversial. Lobectomy and lymphadenectomy are classical surgical approaches for lung cancer. Compared with traditional lung cancer, GGOs, especially pure GGOs show relatively benign biological behavior. It is not currently clear whether lobectomy is still necessary to achieve a “radical” resection of GGOs. In this respect, a series of studies comparing sublobar resection and lobectomy were published. National Comprehensive Cancer Network (NCCN) guidelines have suggested that sublobar resection could be applied for peripheral nodule ≤ 2 cm with at least one of the following criteria: pure AIS histology; nodule has ≥ 50% ground-glass appearance on CT; radiologic surveillance confirms a long doubling time (≥ 400 days).

Nevertheless, only a few studies have focused on the comparison of different approaches of sublobar resection. Previous researches [7–9] compared segmentectomy and wedge resection for all stage I or IA non-small-cell lung cancer (NSCLC) cases, and their main endpoints were overall survival (OS) and disease-free survival (DFS). For GGOs, especially pure GGOs, OS and DFS make little sense as they consist a relatively benign biological behavior, thus the aim to reserve more pulmonary function when the margin is sufficient may be a more meaningful approach. Since no studies have addressed this subject, the application of segmentectomy and wedge resection in peripheral pure GGOs remain controversial.

Successful lung recruitment maneuvers are closely associated with postoperative pulmonary function recovery. This process is mainly affected by two factors: the negative intrathoracic pressure and the surface area of residual lung. Therefore, for a given volume of removed lung tissue, the surgical approach that could leave more surface area brings more advantage for pulmonary function retention. This paper attempts to theoretically analyze the surface area loss caused by sublobar resection of peripheral small GGOs located in each segment using a three-dimensional rebuild of CT images, in order to provide more evidence for a better choice of surgical approach for sublobar resection.

## Methods

The study used the database of our medical center to analyze CT images of 241 patients with peripheral pure GGO(s) located in different segments. There were 15 patients for each segment (some patients have multiple GGOs in different segments). The images were in CT-DICOM format, had layer thickness ≤ 1.25 mm and were scans from apex pulmonis to basis pulmonis. All patients signed a written informed consent during their hospital stay.

The DICOM images were imported into 3DSlicer software, which rebuilds the complete three-dimensional model from two-dimensional slices, and conducts measurement, labeling, segmentation and other operations on the obtained model. Different segments depending on the tracheal distribution were identified and the volume and surface area of different segments and the whole lung were measured. Next, segmentectomy and wedge resection for GGO in the target segments were simulated, and the area of resected surface was measured.

## Results

For each segment, we select a typical patient to show detailed three-dimensional model and calculation process.

### Right superior lobe, apical segment (RS1)

**Fig. 1:**
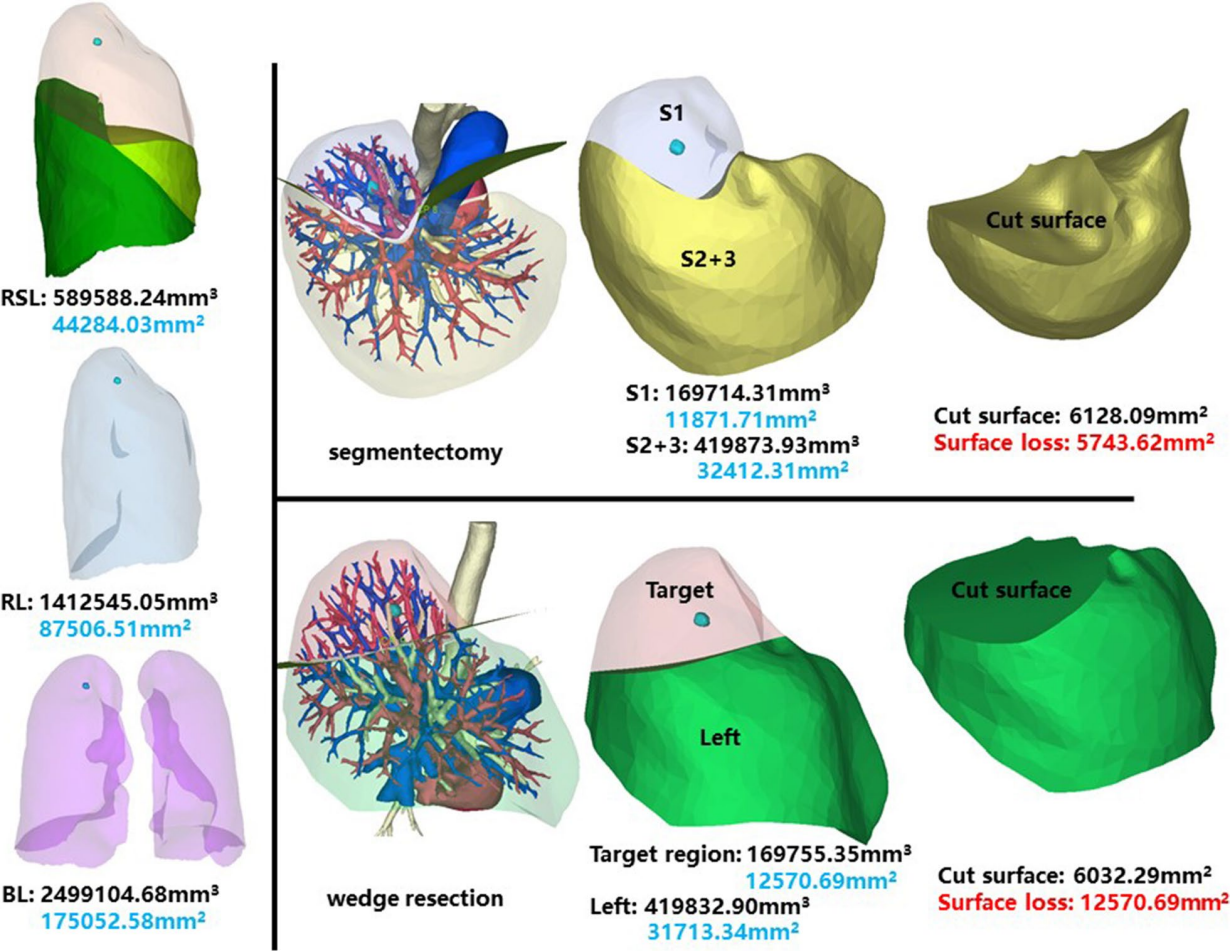
Segmentectomy or wedge resection of RS1. RSL: right superior lung. RL: right lung. BL: bilateral lungs

The whole right superior lobe of patient 1 has a volume of 589,588.24 mm^3^, and a surface area of 44,284.03 mm^2^. The volume and surface area of RS1 are 169,714.31 mm^3^ and 11,871.71 mm^2^, respectively (Fig. 1).

Segmentectomy of RS1 will leave a cut surface of 6128.09 mm^2^, which means a surface loss of 5743.62 mm^2^. As the intersegmental plane is separated with electric scalpel and ultrasonic knife only and no cut surface compression is practical, the 5743.62 mm^2^ is regarded as accessible.

With equal volume of tissue removed, wedge resection will theoretically leave a cut surface of 6032.29 mm^2^. In practice, linear cutters are used, and the whole cut surface is stapled. Thus, the cut surface loss comprises the whole preoperative surface area of the target region, measuring 12,570.69 mm^2^.

### Right superior lobe, posterior segment (RS2)

**Fig. 2:**
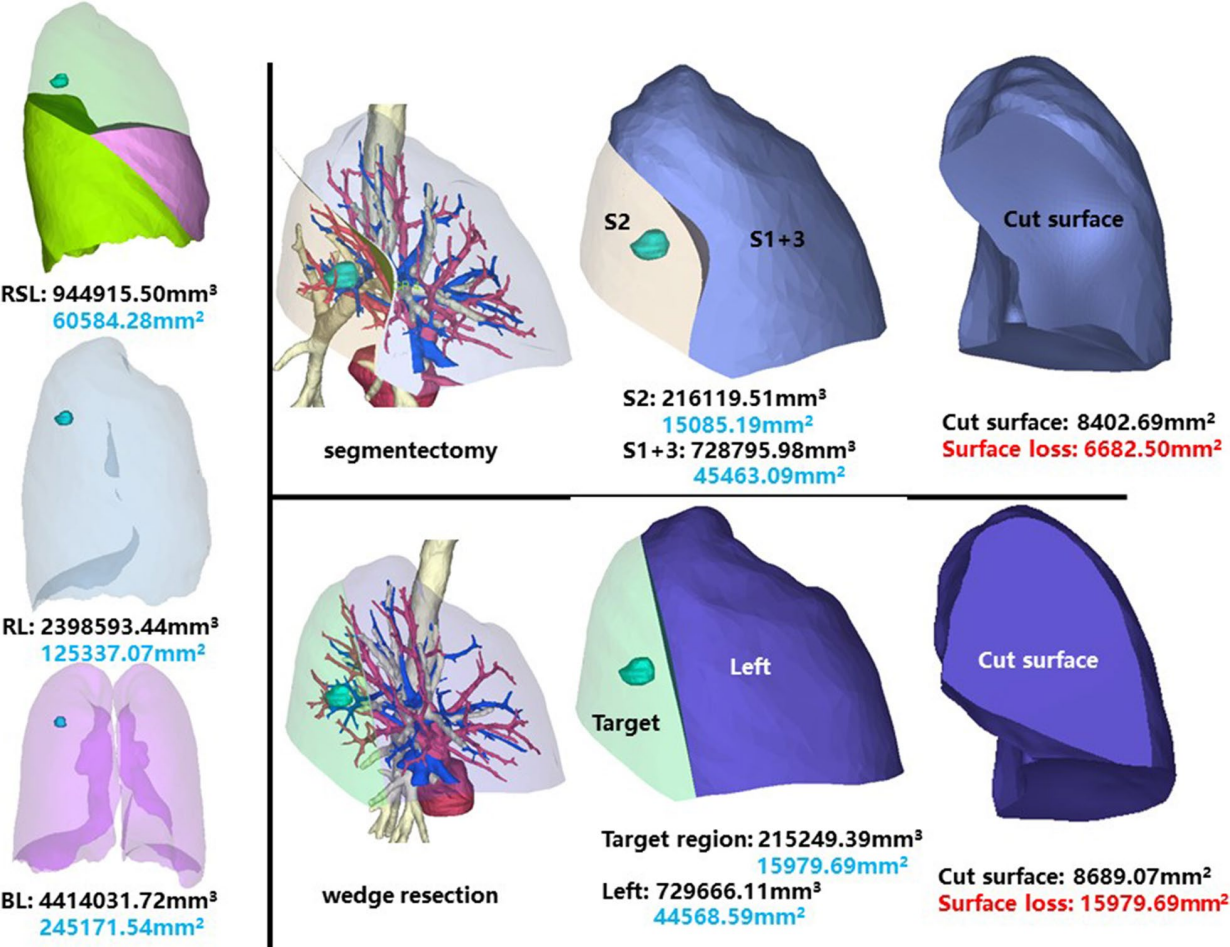
Segmentectomy or wedge resection of RS2

The whole right superior lobe of patient 2 has a volume of 944,915.50 mm^3^, and a surface area of 60,584.28 mm^2^. The volume and surface area of RS2 are 216,119.51 mm^3^ and 15,085.19 mm^2^, respectively (Fig. 2).

Segmentectomy of RS2 will leave a cut surface of 8402.69 mm^2^, which means a surface loss of 6682.50 mm^2^. With equal volume of tissue removed, wedge resection will theoretically leave a cut surface of 8689.07 mm^2^. In practice, the cut surface loss comprises the whole preoperative surface area of the target region, measuring 15,979.69 mm^2^.

### Right superior lobe, anterior segment (RS3)

**Fig. 3:**
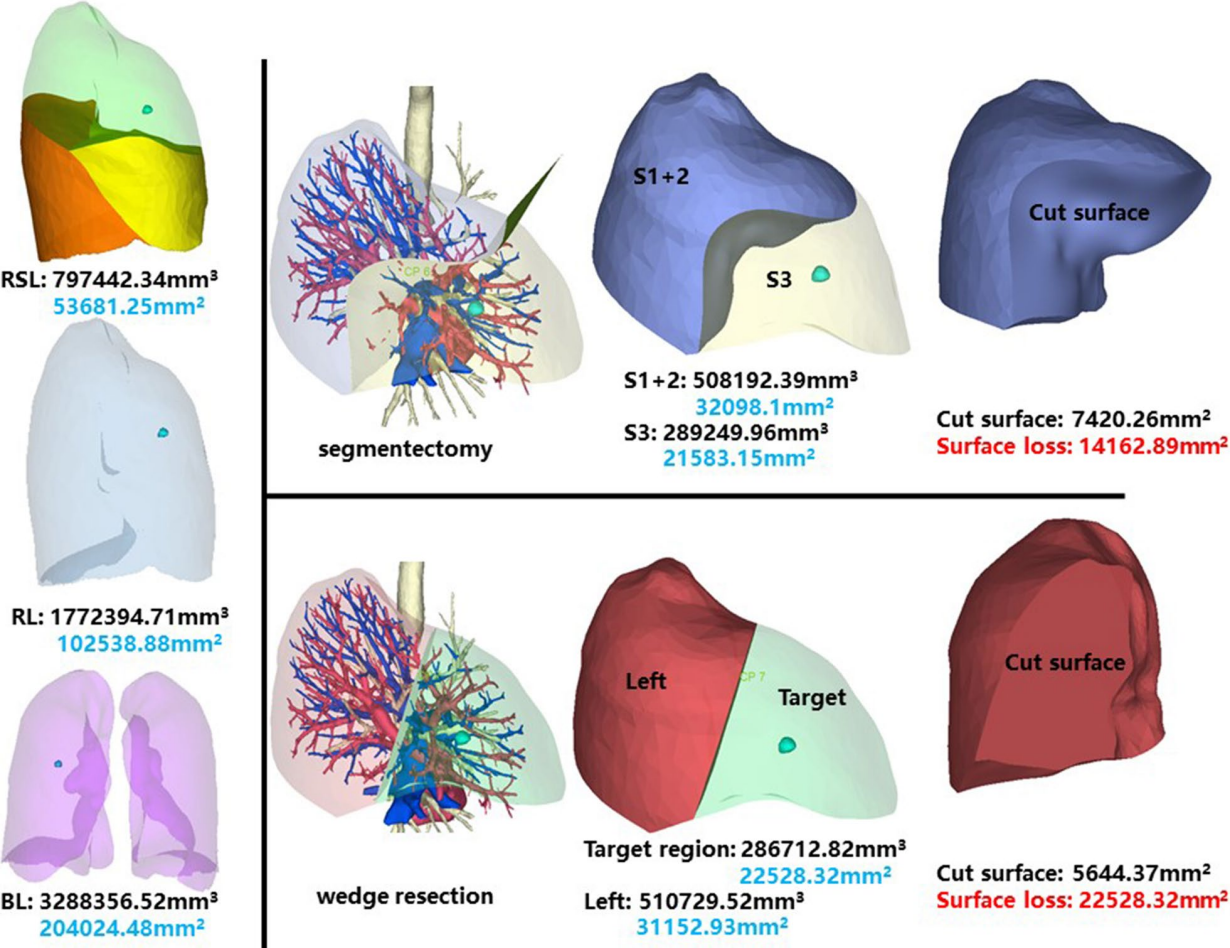
Segmentectomy or wedge resection of RS3

The whole right superior lobe of patient 3 has a volume of 797,442.34 mm^3^, and a surface area of 53,681.25 mm^2^. The volume and surface area of RS3 are 289,249.96 mm^3^ and 21,583.15 mm^2^, respectively (Fig. 3).

Segmentectomy of RS3 will leave a cut surface of 7420.26 mm^2^, which means a surface loss of 14,162.89 mm^2^. With equal volume of tissue removed, wedge resection will theoretically leave a cut surface of 5644.37 mm^2^. In practice, the cut surface loss comprises the whole preoperative surface area of the target region, measuring 22,528.32 mm^2^.

### Right middle lobe, lateral segment (RS4)

**Fig. 4:**
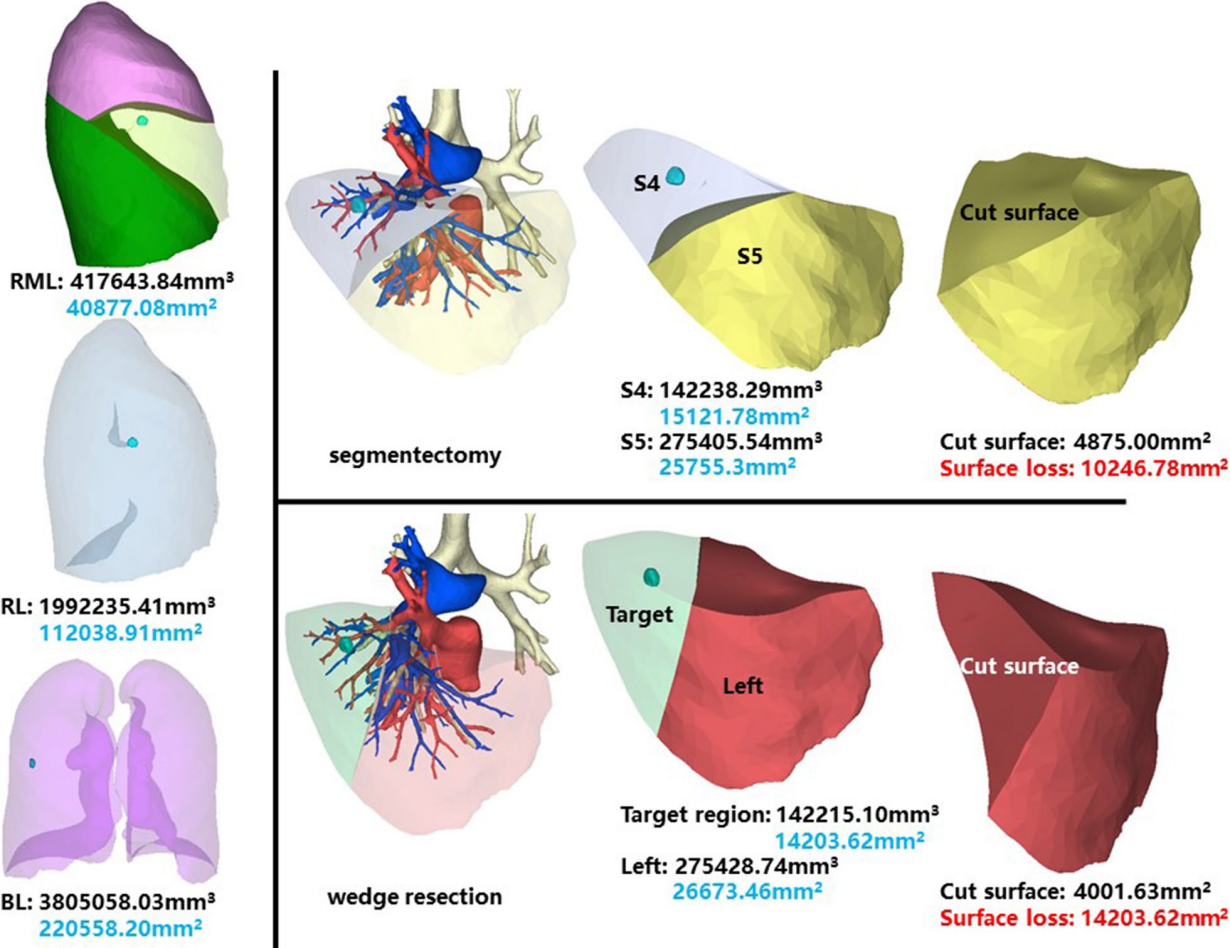
Segmentectomy or wedge resection of RS4. RML: right middle lung

The whole right middle lobe of patient 4 has a volume of 417,643.84 mm^3^, and a surface area of 40,877.08 mm^2^. The volume and surface area of RS4 are 142,238.29 mm^3^ and 15,121.78 mm^2^, respectively (Fig. 4).

Segmentectomy of RS4 will leave a cut surface of 4875.00 mm^2^, which means a surface loss of 10,246.78 mm^2^. With equal volume of tissue removed, wedge resection will theoretically leave a cut surface of 4001.63 mm^2^. In practice, the cut surface loss comprises the whole preoperative surface area of the target region, measuring 14,203.62 mm^2^.

### Right middle lobe, medial segment (RS5)

**Fig. 5:**
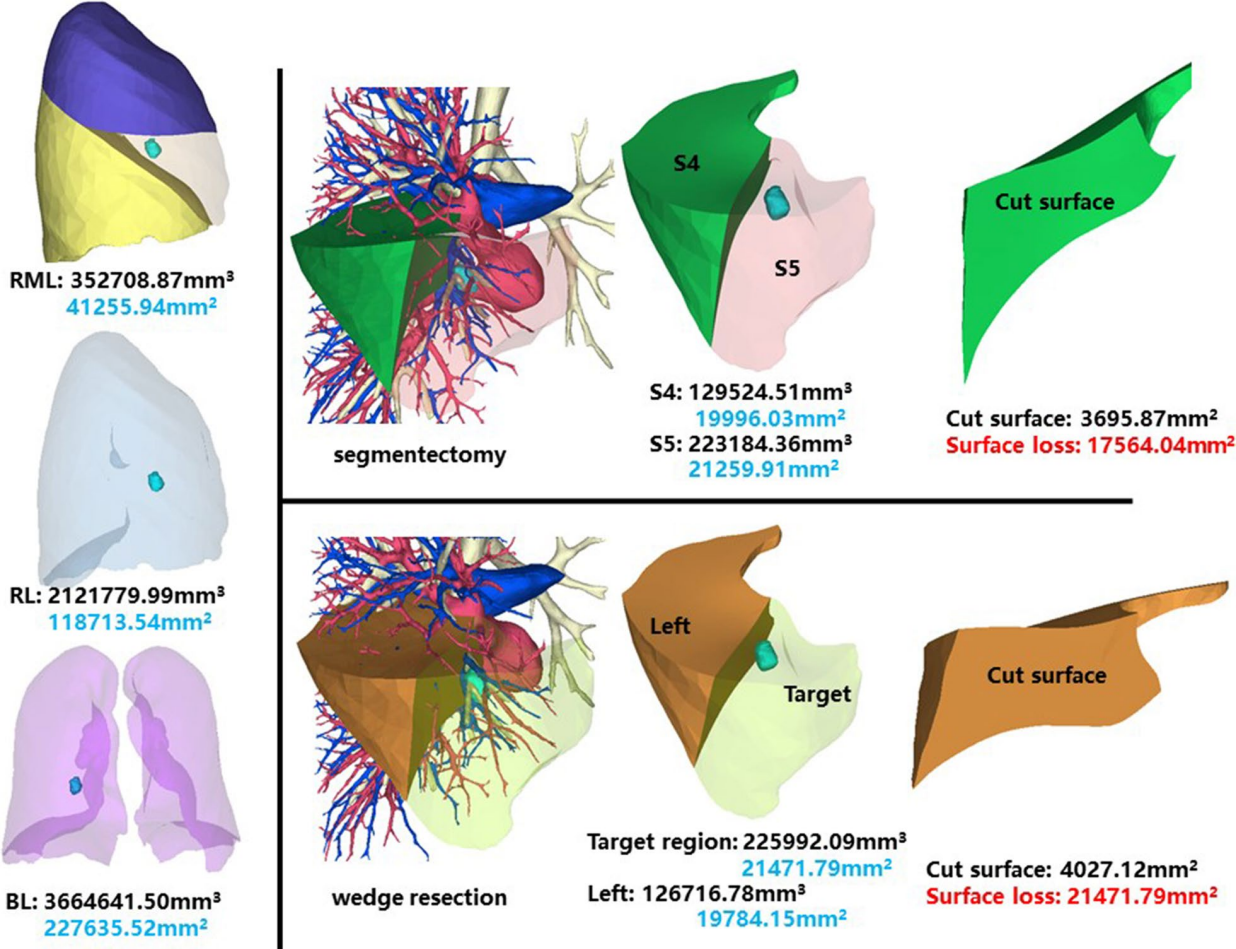
Segmentectomy or wedge resection of RS5

The whole right middle lobe of patient 5 has a volume of 352,708.87 mm^3^, and a surface area of 41,255.94 mm^2^. The volume and surface area of RS5 are 223,184.36 mm^3^ and 21,259.91 mm^2^, respectively (Fig. 5).

Segmentectomy of RS5 will leave a cut surface of 3695.87 mm^2^, which means a surface loss of 17,564.04 mm^2^. With equal volume of tissue removed, wedge resection will theoretically leave a cut surface of 4027.12 mm^2^. In practice, the cut surface loss comprises the whole preoperative surface area of the target region, measuring 21,471.79 mm^2^.

### Right inferior lobe, dorsal segment (RS6)

**Fig. 6:**
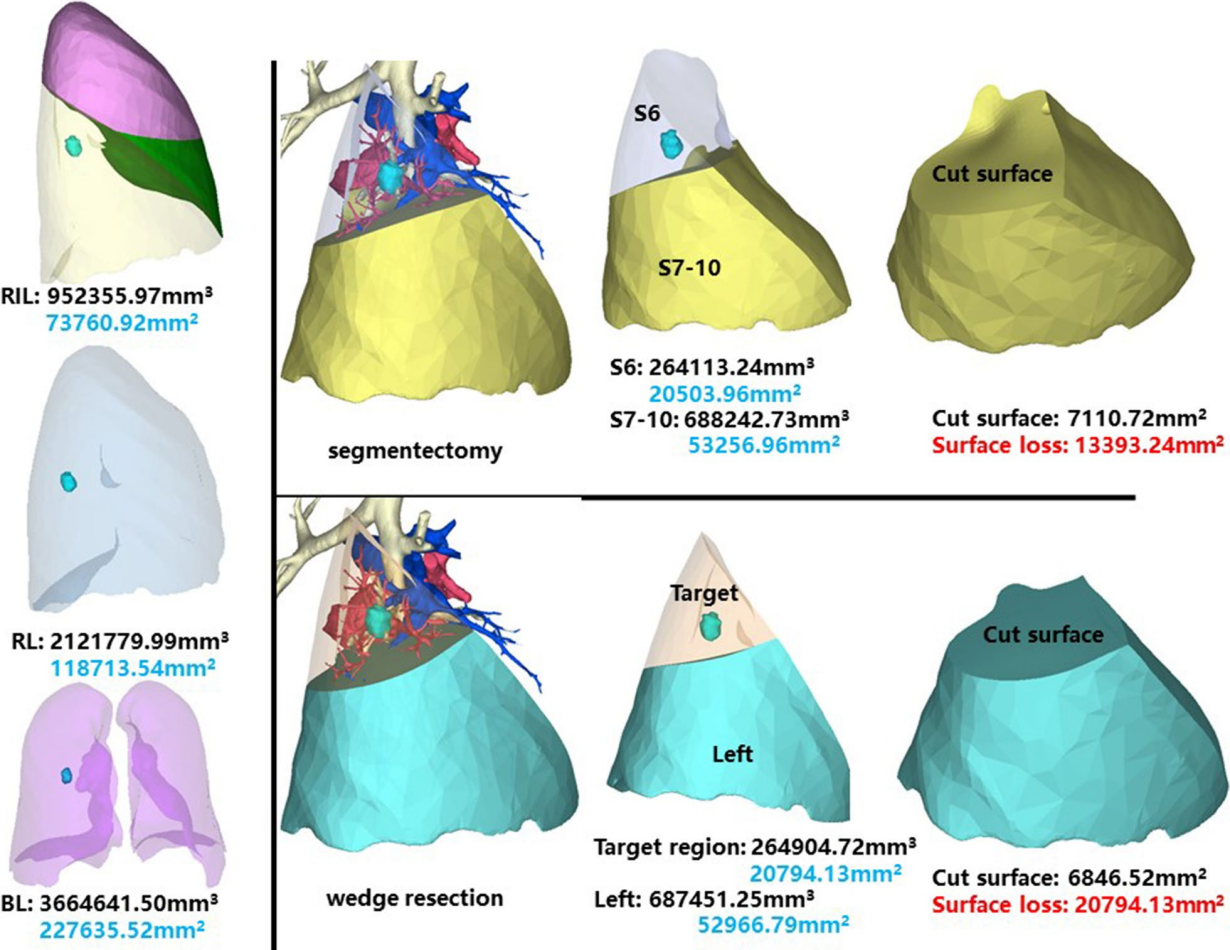
Segmentectomy or wedge resection of RS6. RIL: right inferior lung

This segment is of the same patient as RS5. The whole right inferior lobe of patient 5 has a volume of 952,355.97 mm^3^, and a surface area of 73,760.92 mm^2^. The volume and surface area of RS6 are 264,113.24 mm^3^ and 20,503.96 mm^2^, respectively (Fig. 6).

Segmentectomy of RS6 will leave a cut surface of 7110.72 mm^2^, which means a surface loss of 13,393.24 mm^2^. With equal volume of tissue removed, wedge resection will theoretically leave a cut surface of 6846.52 mm^2^. In practice, the cut surface loss comprises the whole preoperative surface area of the target region, measuring 20,794.13 mm^2^.

### Right inferior lobe, medial basal segment (RS7)

**Fig. 7:**
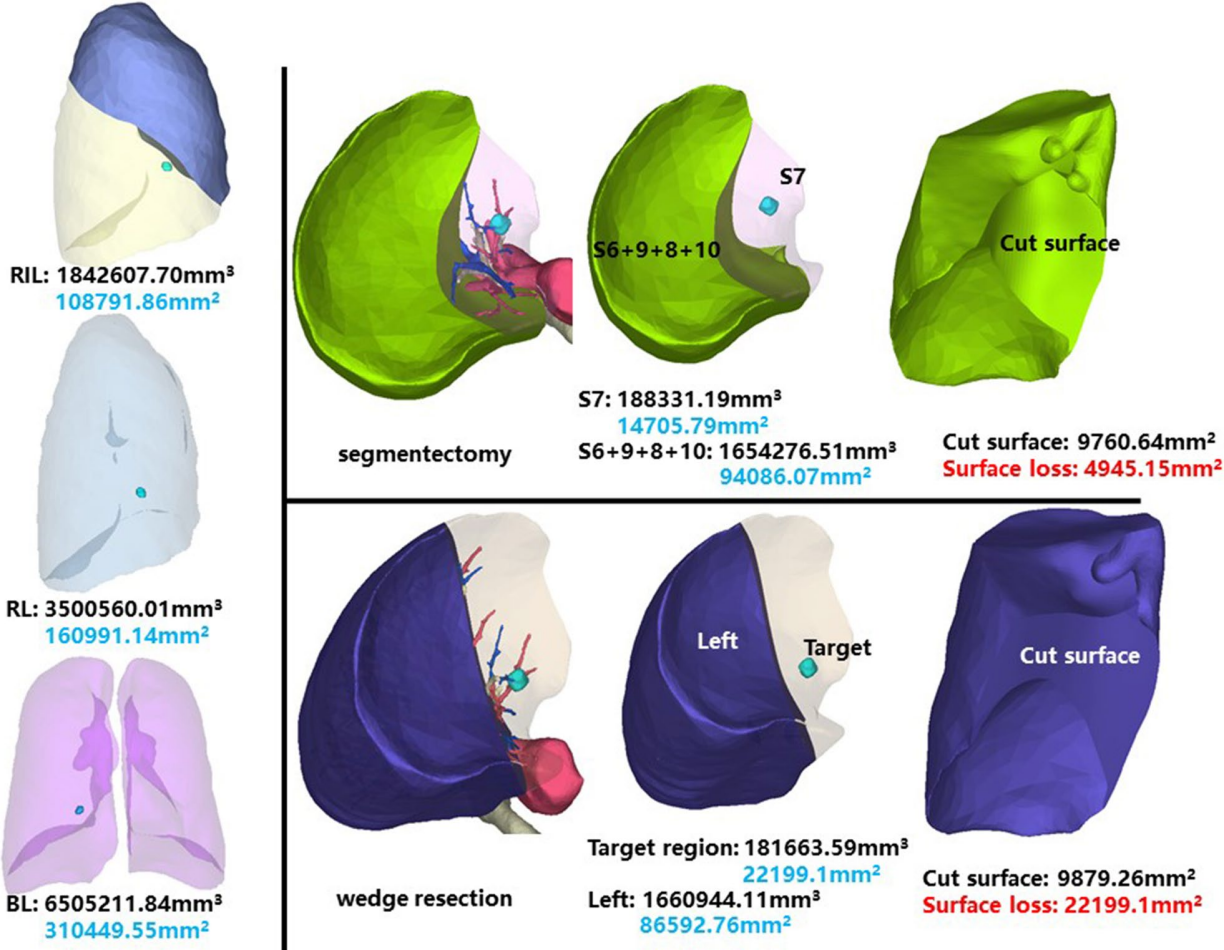
Segmentectomy or wedge resection of RS7

The whole right inferior lobe of patient 6 has a volume of 1,842,607.70 mm^3^, and a surface area of 108,791.86 mm^2^. The volume and surface area of RS7 are 188,331.19 mm^3^ and 14,705.79 mm^2^, respectively (Fig. 7).

Segmentectomy of RS7 will leave a cut surface of 9760.64 mm^2^, which means a surface loss of 4945.15 mm^2^. With equal volume of tissue removed, wedge resection will theoretically leave a cut surface of 9879.26 mm^2^. In practice, the cut surface loss comprises the whole preoperative surface area of the target region, measuring 22,199.1 mm^2^.

### Right inferior lobe, anterior basal segment (RS8)

**Fig. 8:**
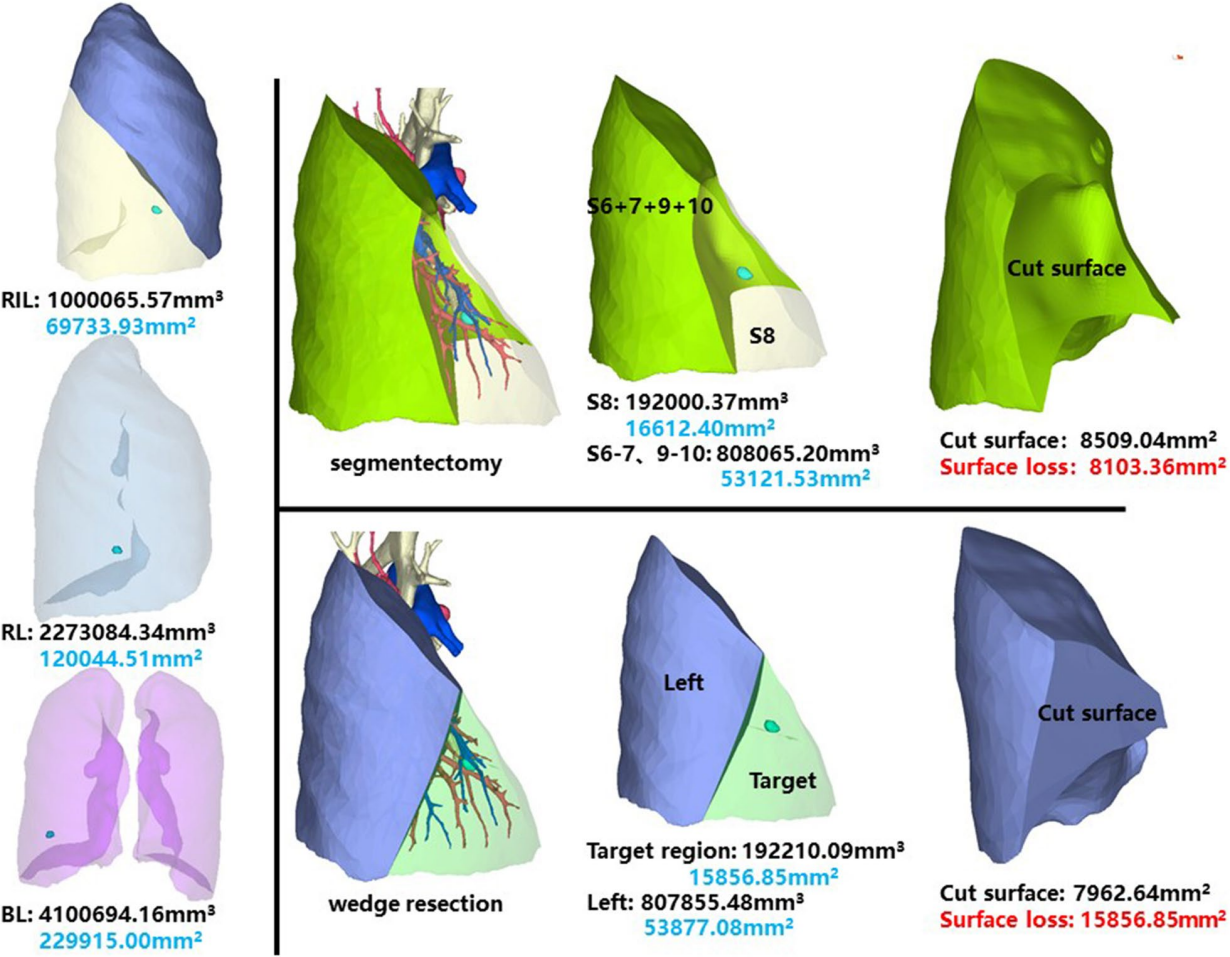
Segmentectomy or wedge resection of RS8

The whole right inferior lobe of patient 7 has a volume of 1,000,065.57 mm^3^, and a surface area of 69,733.93 mm^2^. The volume and surface area of RS8 are 192,000.37 mm^3^ and 16,612.40 mm^2^, respectively (Fig. 8).

Segmentectomy of RS8 will leave a cut surface of 8509.04 mm^2^, which means a surface loss of 8103.36 mm^2^. With equal volume of tissue removed, wedge resection will theoretically leave a cut surface of 7962.64 mm^2^. In practice, the cut surface loss comprises the whole preoperative surface area of the target region, measuring 15,856.85 mm^2^.

### Right inferior lobe, lateral basal segment (RS9)

**Fig. 9:**
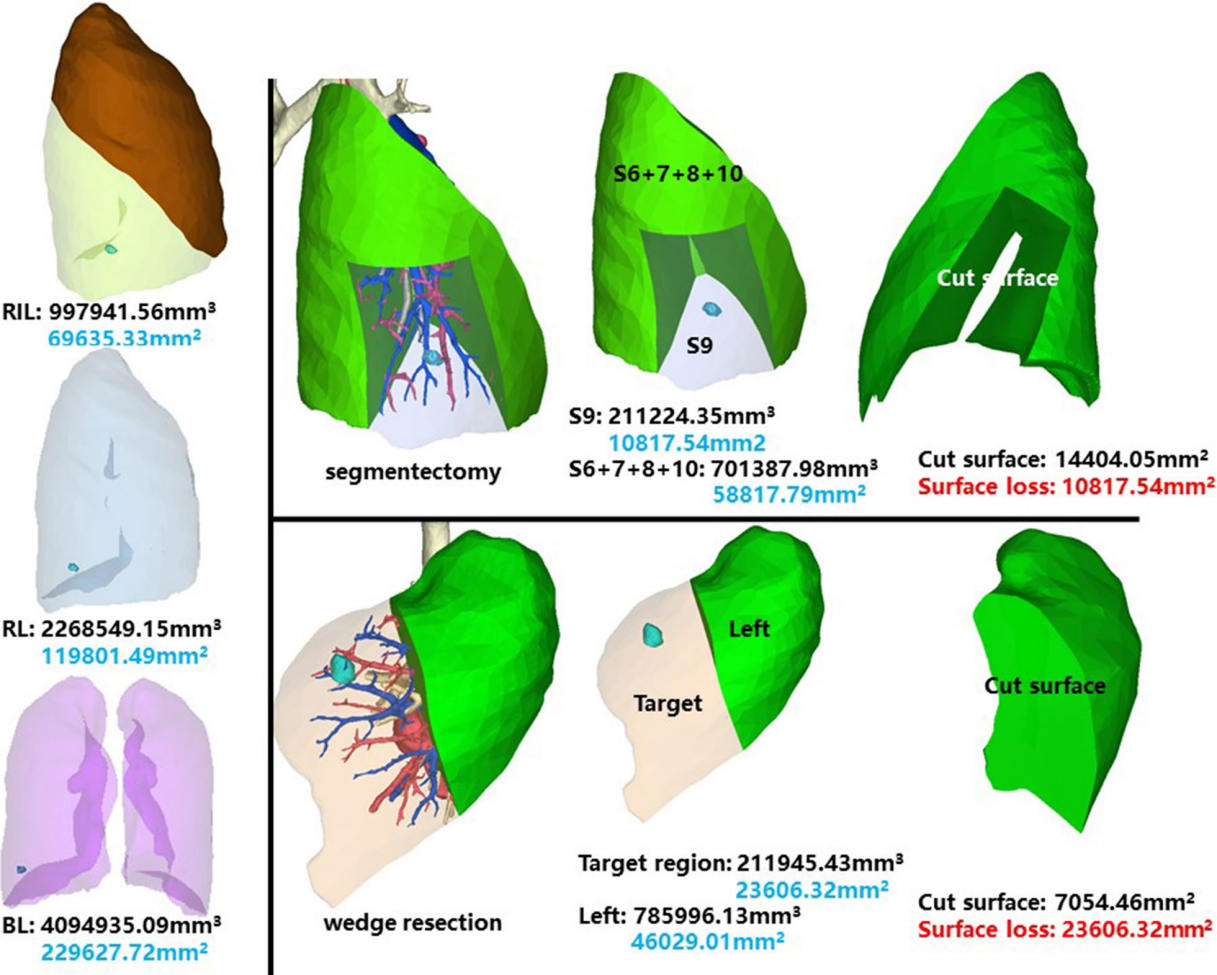
Segmentectomy or wedge resection of RS9

The whole right inferior lobe of patient 8 has a volume of 997,941.56 mm^3^, and a surface area of 69,635.33 mm^2^. The volume and surface area of RS9 are 211,224.35 mm^3^ and 10,817.54 mm^2^, respectively (Fig. 9).

Segmentectomy of RS9 will leave a cut surface of 14,404.05 mm^2^, which means a surface loss of 10,817.54 mm^2^. With equal volume of tissue removed, wedge resection will theoretically leave a cut surface of 7054.46 mm^2^. In practice, the cut surface loss comprises the whole preoperative surface area of the target region, measuring 23,606.32 mm^2^.

### Right inferior lobe, posterior basal segment (RS10)

**Fig. 10:**
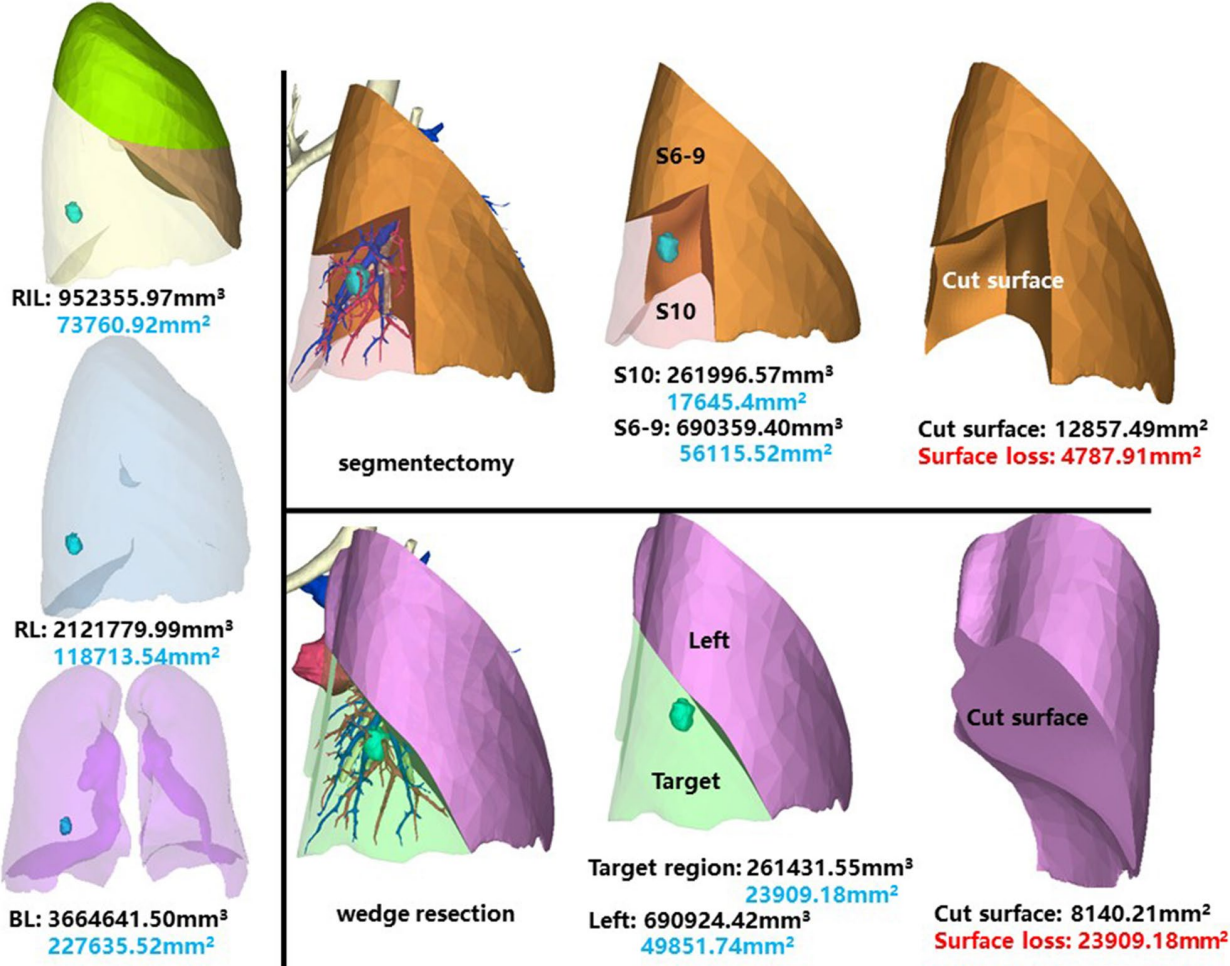
Segmentectomy or wedge resection of RS10

The whole right inferior lobe of patient 9 has a volume of 952,355.97 mm^3^, and a surface area of 73,760.92 mm^2^. The volume and surface area of RS10 are 261,996.57 mm^3^ and 17,645.4 mm^2^, respectively (Fig. 10).

Segmentectomy of RS10 will leave a cut surface of 12,857.49 mm^2^, which means a surface loss of 4787.91 mm^2^. With equal volume of tissue removed, wedge resection will theoretically leave a cut surface of 8140.21 mm^2^. In practice, the cut surface loss comprises the whole preoperative surface area of the target region, measuring 23,909.18 mm^2^.

### Left superior lobe, posterior apical segment (LS1 + 2)

**Fig. 11:**
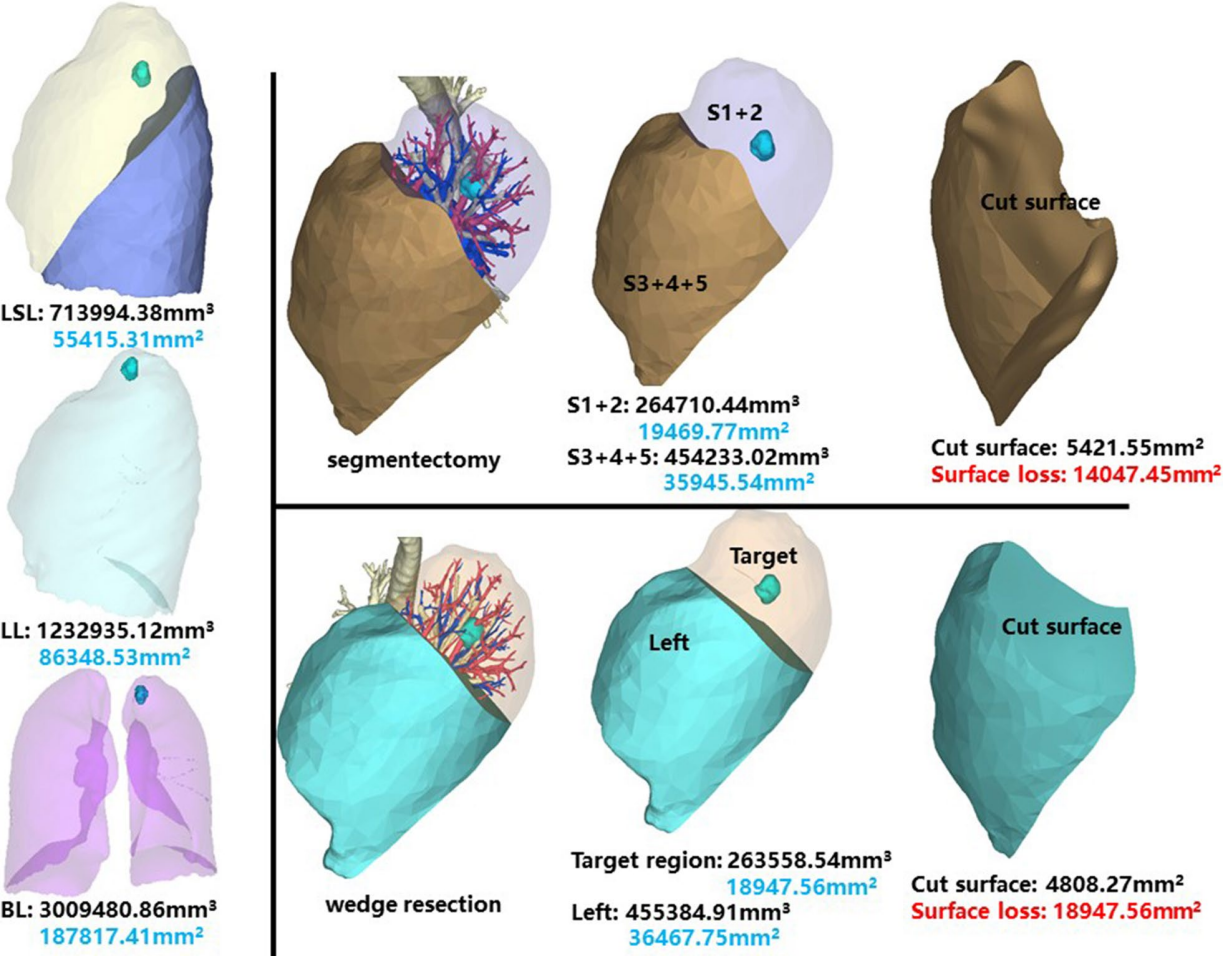
Segmentectomy or wedge resection of LS1 + 2. LSL: left superior lung. LL: left lung

The whole left superior lobe of patient 10 has a volume of 713,994.38 mm^3^, and a surface area of 55,415.31 mm^2^. The volume and surface area of LS1 + 2 are 264,710.44 mm^3^ and 19,469.77 mm^2^, respectively (Fig. 11).

Segmentectomy of LS1 + 2 will leave a cut surface of 5421.55 mm^2^, which means a surface loss of 14,047.45 mm^2^. With equal volume of tissue removed, wedge resection will theoretically leave a cut surface of 4808.27 mm^2^. In practice, the cut surface loss comprises the whole preoperative surface area of the target region, measuring 18,947.56 mm^2^.

### Left superior lobe, anterior segment (LS3)

**Fig. 12:**
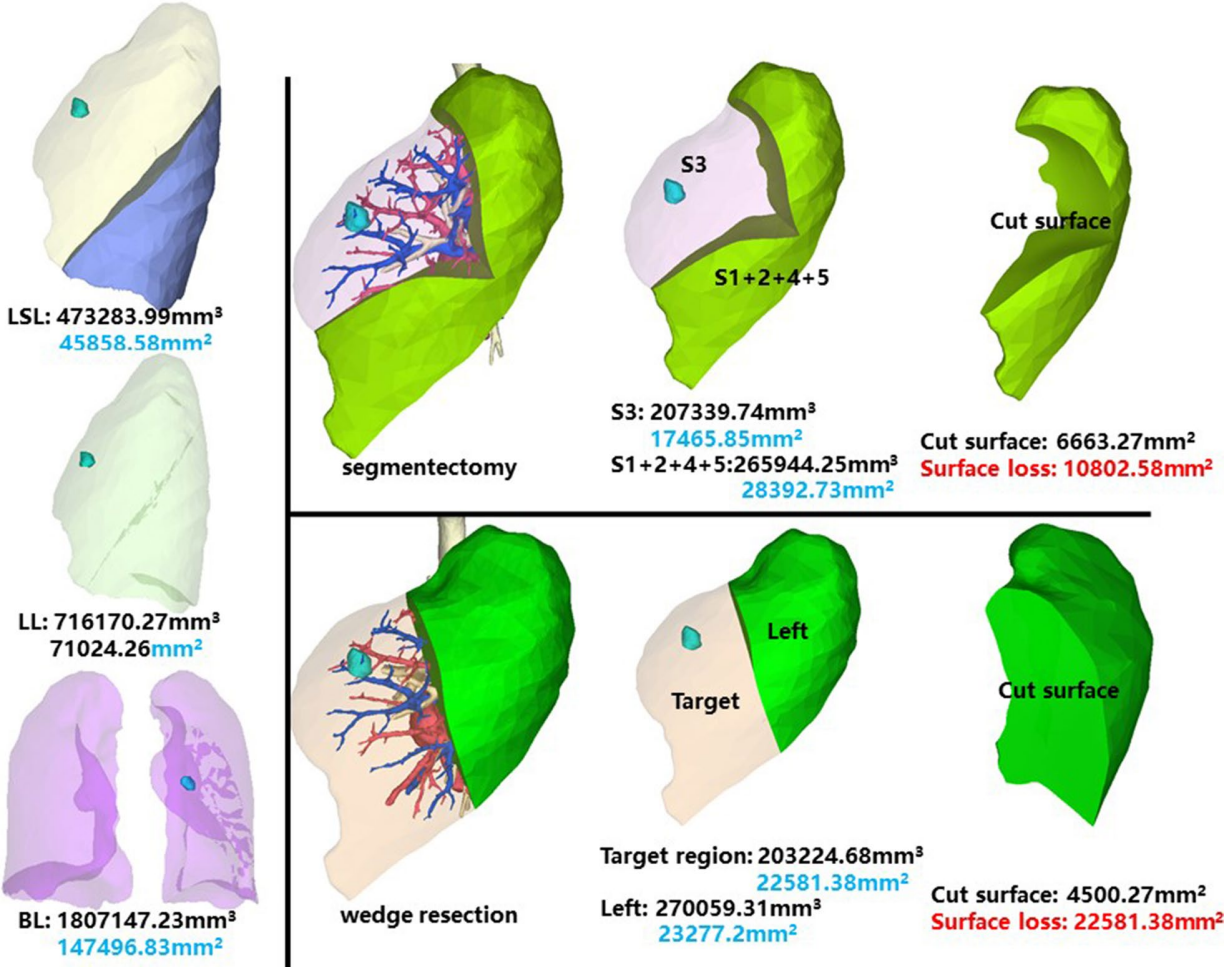
Segmentectomy or wedge resection of LS3

The whole left superior lobe of patient 11 has a volume of 473,283.99 mm^3^, and a surface area of 45,858.58 mm^2^. The volume and surface area of LS3 are 207,339.74 mm^3^ and 17,465.85 mm^2^, respectively (Fig. 12).

Segmentectomy of LS3 will leave a cut surface of 6663.27 mm^2^, which means a surface loss of 10,802.58 mm^2^. With equal volume of tissue removed, wedge resection will theoretically leave a cut surface of 4500.27 mm^2^. In practice, the cut surface loss comprises the whole preoperative surface area of the target region, measuring 22,581.38 mm^2^.

### Left superior lobe, lingual segment (LS4 + 5)

**Fig. 13:**
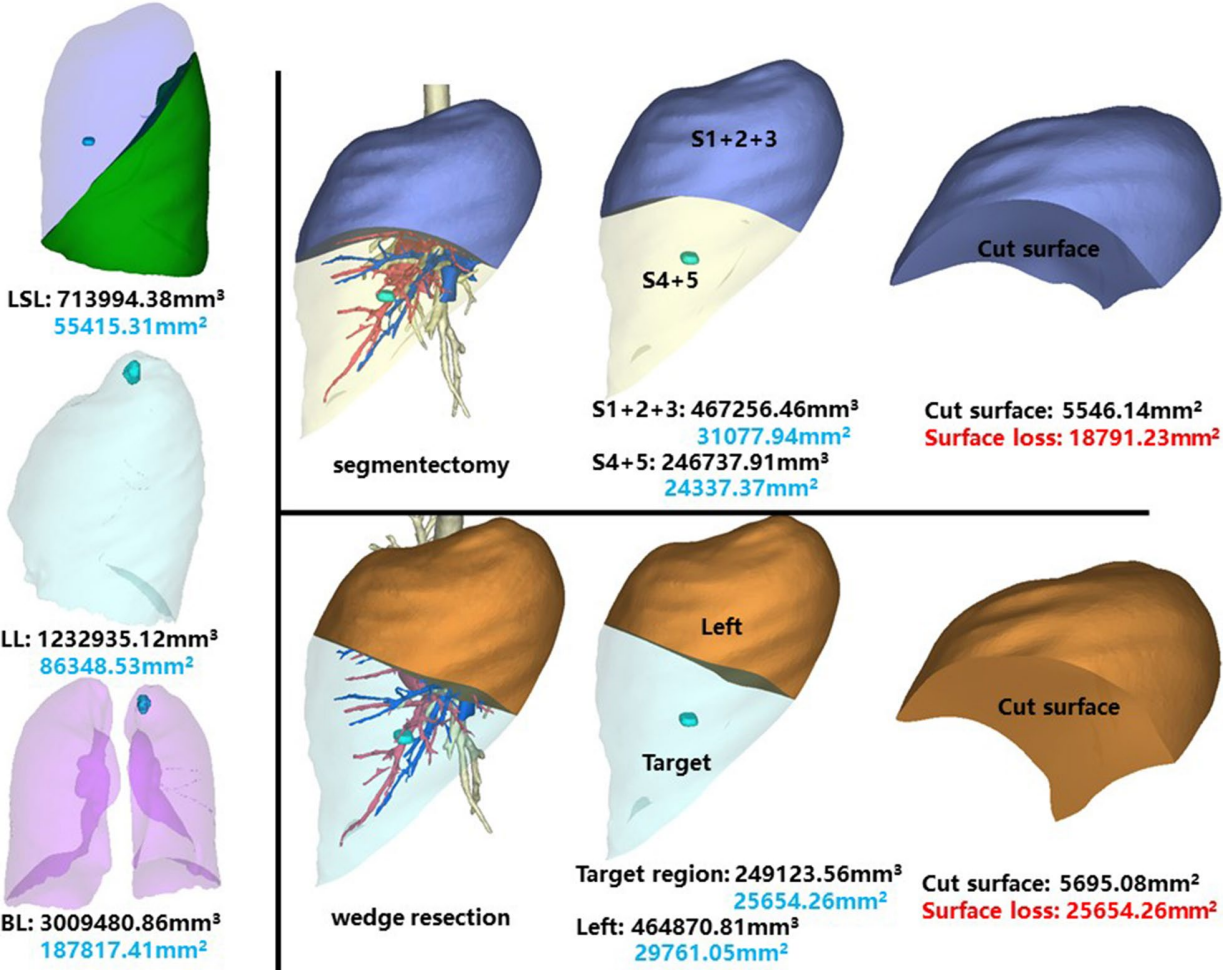
Segmentectomy or wedge resection of LS4 + 5

This segment is of the same patient as LS1 + 2. The volume and surface area of LS4 + 5 are 246,737.91 mm^3^ and 24,337.37 mm^2^, respectively (Fig. 13).

Segmentectomy of LS4 + 5 will leave a cut surface of 5546.14 mm^2^, which means a surface loss of 18,791.23 mm^2^. With equal volume of tissue removed, wedge resection will theoretically leave a cut surface of 5695.08 mm^2^. In practice, the cut surface loss comprises the whole preoperative surface area of the target region, measuring 25,654.26 mm^2^.

### Left inferior lobe, dorsal segment (LS6)

**Fig. 14:**
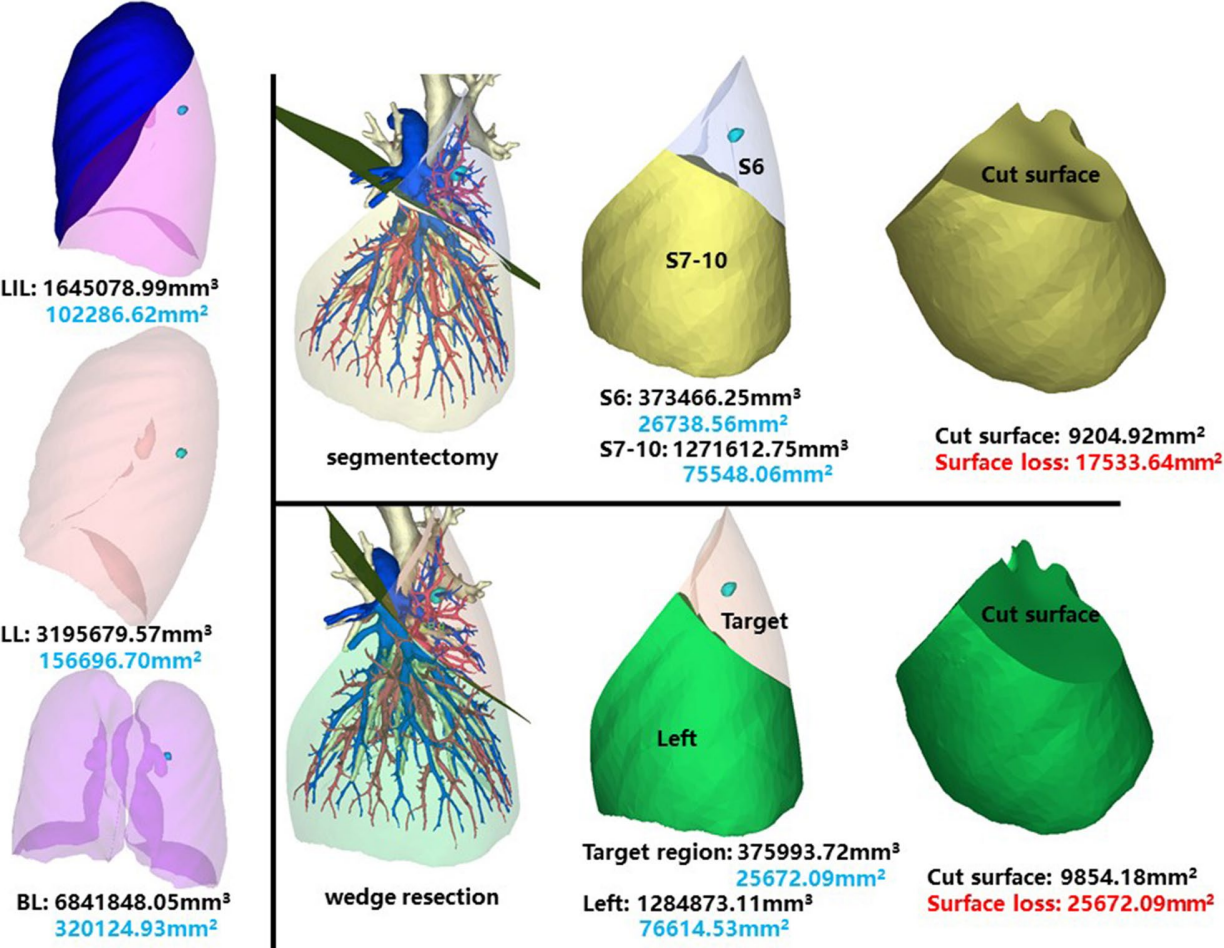
Segmentectomy or wedge resection of LS6. LIL: left inferior lung

The whole left inferior lobe of patient 12 has a volume of 1,645,078.99 mm^3^, and a surface area of 102,286.62 mm^2^. The volume and surface area of LS6 are 373,466.25 mm^3^ and 26,738.56 mm^2^, respectively (Fig. 14).

Segmentectomy of LS6 will leave a cut surface of 9204.92 mm^2^, which means a surface loss of 17,533.64 mm^2^. With equal volume of tissue removed, wedge resection will theoretically leave a cut surface of 9854.18 mm^2^. In practice, the cut surface loss comprises the whole preoperative surface area of the target region, measuring 25,672.09 mm^2^.

### Left inferior lobe, medial anterior basal segment (LS7 + 8)

**Fig. 15:**
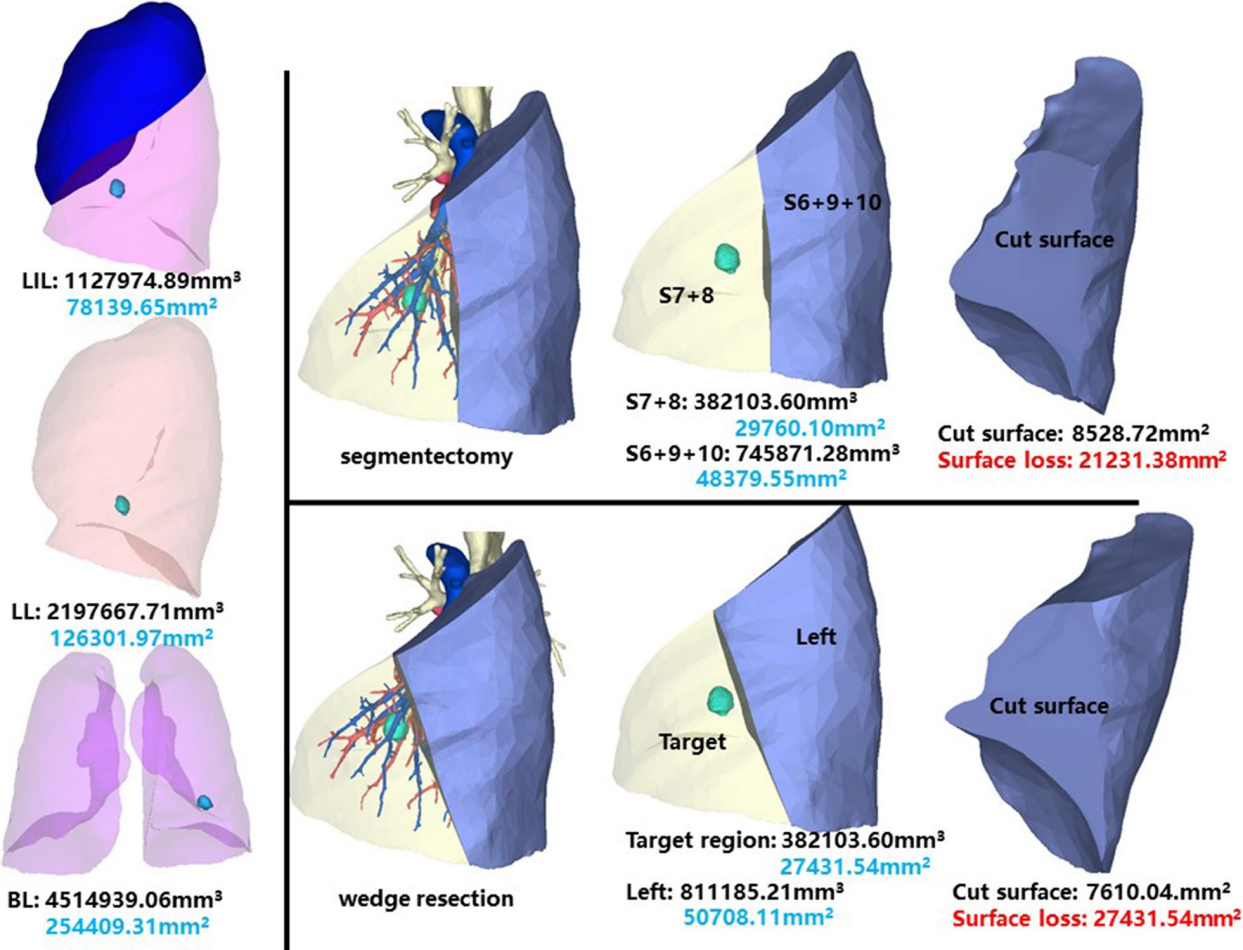
Segmentectomy or wedge resection of LS7 + 8

The whole left inferior lobe of patient 13 has a volume of 1,127,974.89 mm^3^, and a surface area of 78,139.65 mm^2^. The volume and surface area of LS7 + 8 are 382,103.60 mm^3^ and 29,760.10 mm^2^, respectively (Fig. 15).

Segmentectomy of LS7 + 8 will leave a cut surface of 8528.72 mm^2^, which means a surface loss of 21,231.38 mm^2^. With equal volume of tissue removed, wedge resection will theoretically leave a cut surface of 7610.04 mm^2^. In practice, the cut surface loss comprises the whole preoperative surface area of the target region, measuring 27,431.54 mm^2^.

### Left inferior lobe, lateral anterior basal segment (LS9)

**Fig. 16:**
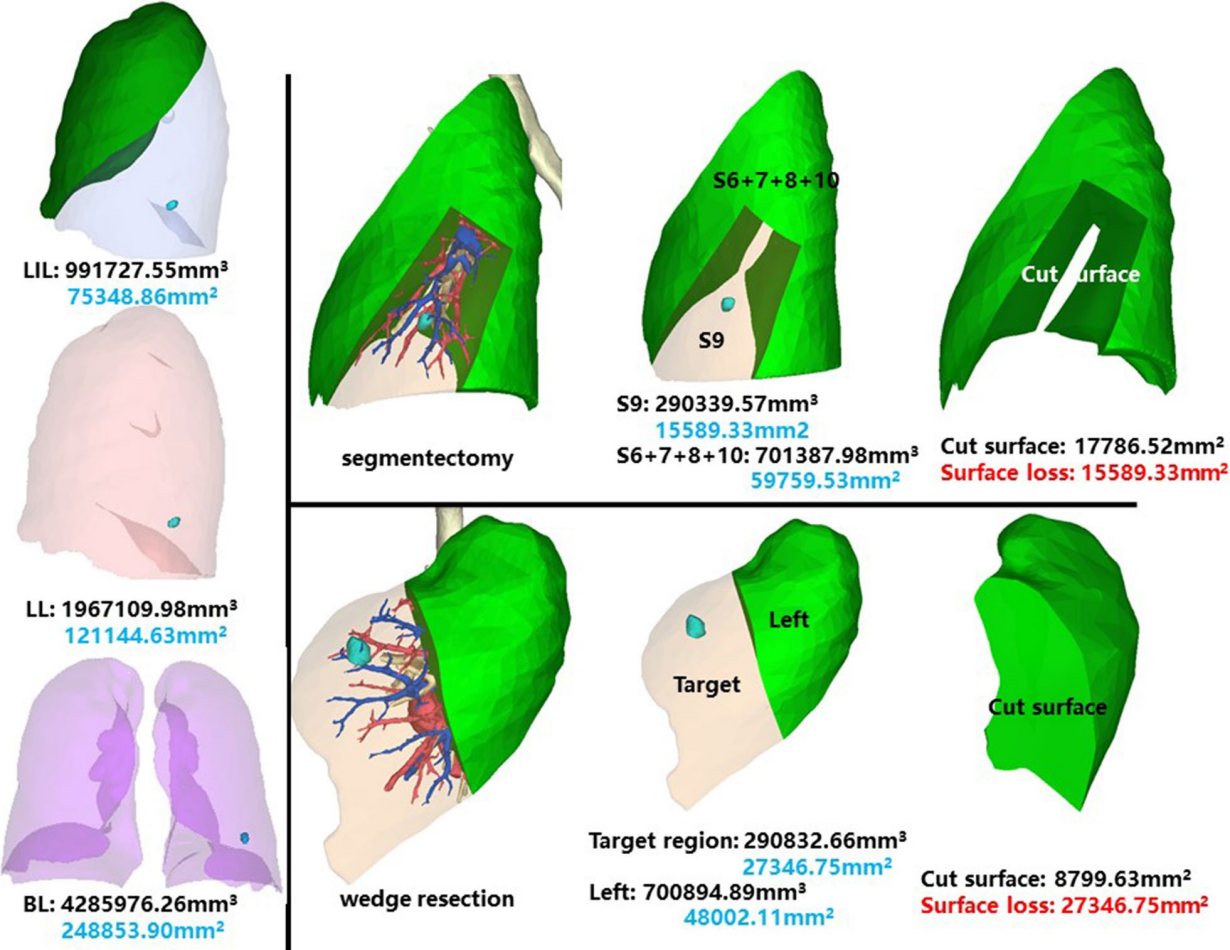
Segmentectomy or wedge resection of LS9

The whole left inferior lobe of patient 14 has a volume of 991,727.55 mm^3^, and a surface area of 75,348.86 mm^2^. The volume and surface area of LS9 are 290,339.57 mm^3^ and 15,589.33 mm^2^, respectively (Fig. 16).

Segmentectomy of LS9 will leave a cut surface of 17,786.52 mm^2^, which means a surface loss of 15,589.33 mm^2^. With equal volume of tissue removed, wedge resection will theoretically leave a cut surface of 8799.63 mm^2^. In practice, the cut surface loss comprises the whole preoperative surface area of the target region, measuring 27,346.75 mm^2^.

### Left inferior lobe, posterior basal segment (LS10)

**Fig. 17:**
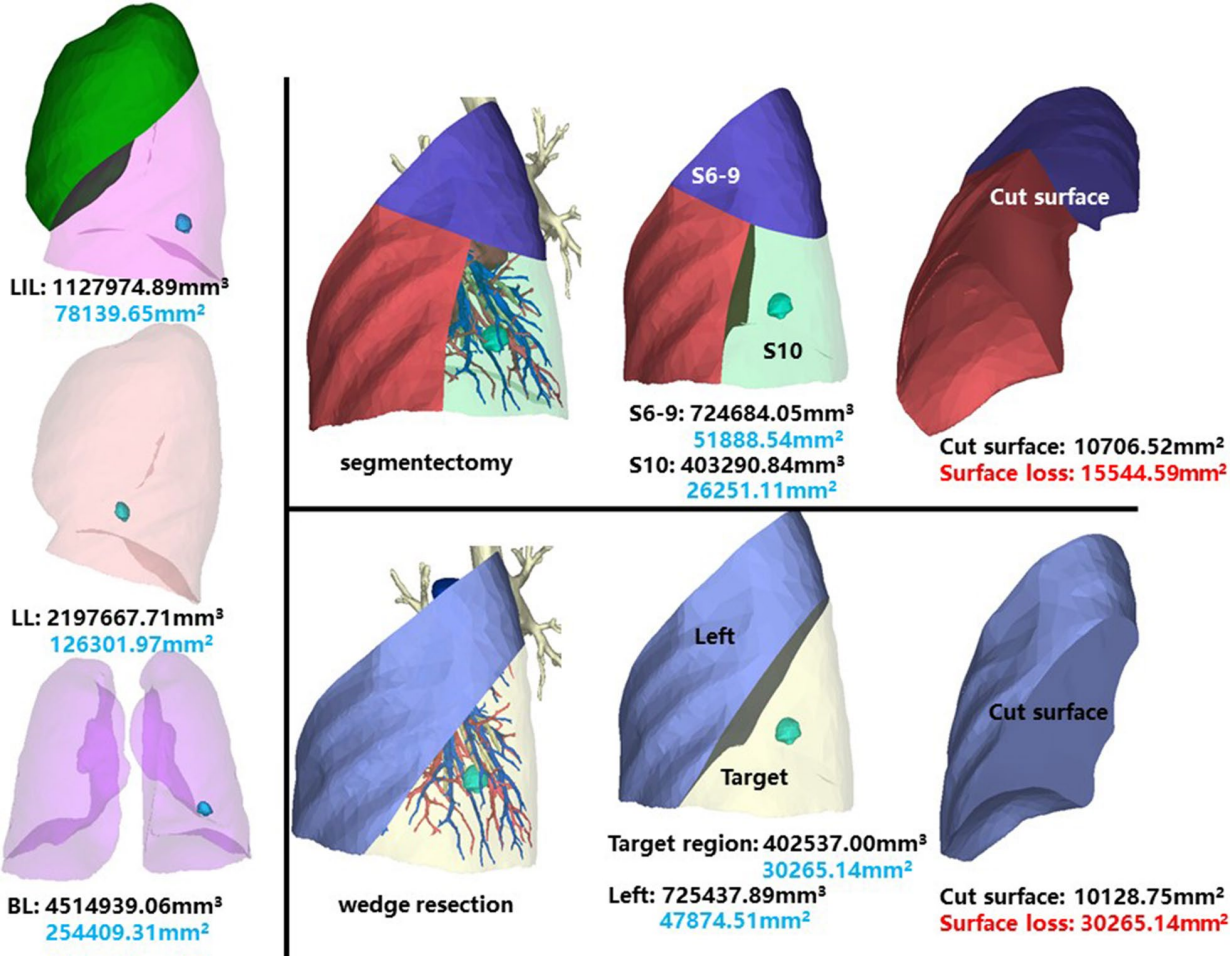
Segmentectomy or wedge resection of LS10

This segment is of the same patient as LS7 + 8. The volume and surface area of LS10 are 403,290.84 mm^3^ and 26,251.11 mm^2^, respectively (Fig. 17).

Segmentectomy of LS10 will leave a cut surface of 10,706.52 mm^2^, which means a surface loss of 15,544.59 mm^2^. With equal volume of tissue removed, wedge resection will theoretically leave a cut surface of 10,128.75 mm^2^. In practice, the cut surface loss comprises the whole preoperative surface area of target region, measuring 30,265.14 mm^2^.

For each of all of the segments, the mean difference value of the surface loss of all segments after simulation of segmentectomy and wedge resection of the 241 patients was calculated (Table 1). After converting the result into relative value of whole pulmonary surface area, it was found that both segments of right middle lobe showed an unobvious surface loss gap between the two approaches, followed by LS1 + 2 and LS7 + 8. On the contrary, wedge resection performed in RS8, RS7, RS10, LS3, LS10. RS9 and LS9 segments may lead to much higher surface area loss than segmentectomy, as compared with other segments.

**Table 1:** The surface loss of all segments after segmentectomy and wedge resection, and their difference

Segment	Total pulmonary surface area (mm^2^)	Surface loss of segmentectomy (mm^2^)	Surface loss of wedge resection (mm^2^)	Difference value (mm^2^)	Difference value/total pulmonary surface area (%)
RS4	254,751.29	12,356.17	15,503.65	3147.48	1.24
RS5	253,657.46	16,348.54	20,586.35	4237.81	1.67
LS1 + 2	241,265.57	15,214.75	19,632.18	4417.43	1.83
LS7 + 8	268,562.24	23,451.58	28,412.68	4961.1	1.85
LS6	271,421.32	14,971.07	21,751.2	6780.13	2.50
RS6	238,895.42	14,542.49	21,256.85	6714.36	2.81
RS3	241,235.59	14,582.27	21,473.56	6891.29	2.86
RS1	256,916.43	6428.45	14,304.08	7875.63	3.07
LS4 + 5	249,867.79	22,461.04	30,466.32	8005.28	3.20
RS2	245,876.66	7005.53	15,861.24	8855.71	3.60
LS9	265,233.24	16,231.25	26,351.85	10,120.6	3.82
RS9	266,705.61	11,481.47	22,744.04	11,262.57	4.22
LS10	274,456.43	14,566.02	27,970.87	13,404.85	4.88
LS3	236,481.09	14,928.14	28,601.18	13,673.04	5.78
RS10	261,171.69	5268.19	21,127.29	15,859.1	6.07
RS7	274,986.12	4896.48	21,669.6	16,773.12	6.10
RS8	228,569.78	11,356.23	25,523.52	14,167.29	6.20

## Discussion

Sublobar resection was previously the only alternative for patients that could not tolerate lobectomy. A prospective, multicenter randomized clinical trial showed that sublobar resection did not show any differences in perioperative complications, mortality, and postoperative pulmonary function after lobectomy, but the recurrence rate was three times as high as that after lobectomy [10]. A different prospective, multicenter non-randomized clinical trial reported similar results, in that the sublobar resection group had a higher local recurrence rate, and the lobectomy group featured higher 5-year OS [11]. These well-known researches established lobectomy as a “golden standard” of surgical treatment for NSCLC. In recent years, with a growing number of patients presenting NSCLC with low potential malignancy, sublobar resection is returning to practice. In clinical work, most pure GGOs fit the NCCN criteria: pure AIS histology, nodule has ≥ 50% ground-glass appearance on CT, and radiologic surveillance confirms a long doubling time (≥ 400 days). Therefore, the applicability of sublobar resection in patients with pure GGOs is widely accepted.

Regrettably, no studies have provided guidance on the choice of surgical approach for sublobar resection of pure GGOs, as pulmonary function preservation has priority in pure GGO patients, unlike in those with solid nodules. For pure GGOs beneath the visceral pleura, simple wedge resection could achieve the treatment goal; for pure GGOs located near the segmental portal, segmentectomy is necessary; whereas for other peripheral pure GGOs, it is difficult to compare the resected pulmonary volume when margin is ensured. Therefore, we set a relatively “standard” model to make the comparisons.

Our model was set up based on following assumptions: a. segmentectomy and wedge resection affect equal pulmonary volumes; b. only power equipments (electric scalpel and ultrasonic knife) are used to separate the intersegmental plane (as linear cutters may be used in practice, this model only considers a fully stretched status of intersegmental plane); c. there is no adhesion in the thorax and the interlobar fissure develops well; d. the anatomical structure and volume distribution of all segments are standard without variation.

According to the three-dimensional reconstruction of CT images, different segments show significantly different surface loss values between segmentectomy and wedge resection. Regarding surface loss, it is the spatial structure and neighbouring relations of each segment that matter the most. Irregular and large-sized cut surface (e.g., RS10, LS3 and LS10) means more tissue compression needed to perform wedge resection with linear cutters, which corresponds to more surface loss. For a cut surface that is small and plane (e.g., RS4, RS5 and LS7 + 8), wedge sections may be appropriate as surface loss is acceptable compared with segmentectomy. Surface loss has adverse effects on pulmonary recruitment, and induce postoperative pulmonary atelectasis, which is commonly seen in CT images after wedge resections, especially the wedge resection of S10. Segmentectomy can help to solve this issue. Following the segmentectomy of S10, well re-tensioned lungs can be seen without pulmonary atelectasis and shrivel in CT images, especially when the intersegmental plane is separated by power equipments only. Therefore, for segments RS4, RS5, LS1 + 2, LS6 and LS7 + 8, wedge resection is suggested; for segments RS1, RS2, RS6, RS8 and LS4 + 5, segmentectomy is suggested, and deep wedge resection should be avoided for segments RS7, RS9, RS10, LS3 and LS10, as a stapled cut surface will have huge impact on postoperative pulmonary recruitment.

In addition, most lung tissues removed by wedge resection are peripheral, which contain more alveolar tissue for gas exchange, while residual lung tissue has more airways and blood vessels for air and blood delivery. On the other hand, segmentectomy-resected alveolar tissue and the corresponding airways and blood vessels form a functional unit as a whole, and the residual lung tissue is still a unit of complete respiratory function. Therefore, wedge resection with disproportional removal of tissue types will have a more adverse impact on air exchange function compared with segmentectomy.

The present study has certain limitations and features worth noting. Firstly, it only targets pure GGOs that are peripheral, but still show a certain distance from the surface, which need a “deep” wedge resection to ensure the margin. Surgical procedures for such lesions are the most complex ones. Secondly, the volume of each segment differs between individuals, thus the volume and surface area of our model is not able to fit all situations. Thirdly, as intrathoracic adhesions, undeveloped interlobar fissure and hilar area are not correlated with pulmonary recruitment, they were not involved in our model. Fourthly, many thoracic surgeons use a stapler to separate part or all of the intersegmental plane, which still results in surface loss and neighboring segment compression. Therefore, from the perspective of pulmonary function reserve, it is suggested to separate the intersegmental plane in segmentectomy using power equipment only. On some occasions, wedge resection may be performed as nonlinear, which is not discussed in this article, either.

The conclusions drawn in this paper still need to be verified. Therefore, we look forward to the reporting of more convincing and robust data that could eventually resolve the controversy between segmentectomy and wedge resection.

## Conclusion

Wedge resection is suggested for some certain segments,such as RS4, RS5, LS1 + 2 and LS7 + 8, whereas segmentectomy is advised for segments RS1, LS4 + 5 and RS2. Meanwhile, deep wedge resection should be avoided for segments RS8, RS7, RS10, LS3, LS10. RS9 and LS9, in order to preserve a larger lung surface area.

## Data Availability

All data is contained within the manuscript and its additional files.

## Abbreviations

GGO: Ground-glass opacities
CT: Computed tomography
AAH: Atypical adenomatous hyperplasia
AIS: Adenocarcinoma in situ
MIA: Or malignancies such as minimally invasive adenocarcinoma
LPA: Lepidic-predominant invasive adenocarcinomas
NCCN: National Comprehensive Cancer Network
NSCLC: Non-small-cell lung cancer
OS: Overall survival
DFS: Disease-free survival
